# Immunotherapy in Treating EGFR-Mutant Lung Cancer: Current Challenges and New Strategies

**DOI:** 10.3389/fonc.2021.635007

**Published:** 2021-05-25

**Authors:** Kenneth K. W. To, Winnie Fong, William C. S. Cho

**Affiliations:** ^1^ School of Pharmacy, Faculty of Medicine, The Chinese University of Hong Kong, Hong Kong, Hong Kong; ^2^ Department of Clinical Oncology, Queen Elizabeth Hospital, Kowloon, Hong Kong

**Keywords:** targeted therapy, non-small cell lung cancer, immunotherapy, PD-1, PD-L1, EGFR mutation, tyrosine kinase inhibitor

## Abstract

Lung cancer is the leading cause of cancer-related deaths worldwide. Immune checkpoint inhibitors, including monoclonal antibodies against programmed death-1 (PD-1) and programmed death ligand-1 (PD-L1), have dramatically improved the survival and quality of life of a subset of non-small cell lung cancer (NSCLC) patients. Multiple predictive biomarkers have been proposed to select the patients who may benefit from the immune checkpoint inhibitors. EGFR-mutant NSCLC is the most prevalent molecular subtype in Asian lung cancer patients. However, patients with EGFR-mutant NSCLC show poor response to anti-PD-1/PD-L1 treatment. While small-molecule EGFR tyrosine kinase inhibitors (TKIs) are the preferred initial treatment for EGFR-mutant NSCLC, acquired drug resistance is severely limiting the long-term efficacy. However, there is currently no further effective treatment option for TKIs-refractory EGFR-mutant NSCLC patients. The reasons mediating the poor response of EGFR-mutated NSCLC patients to immunotherapy are not clear. Initial investigations revealed that EGFR-mutated NSCLC has lower PD-L1 expression and a low tumor mutational burden, thus leading to weak immunogenicity. Moreover, the use of PD-1/PD-L1 blockade prior to or concurrent with osimertinib has been reported to increase the risk of pulmonary toxicity. Furthermore, emerging evidence shows that PD-1/PD-L1 blockade in NSCLC patients can lead to hyperprogressive disease associated with dismal prognosis. However, it is difficult to predict the treatment toxicity. New biomarkers are urgently needed to predict response and toxicity associated with the use of PD-1/PD-L1 immunotherapy in EGFR-mutated NSCLC. Recently, promising data have emerged to suggest the potentiation of PD-1/PD-L1 blockade therapy by anti-angiogenic agents and a few other novel therapeutic agents. This article reviews the current investigations about the poor response of EGFR-mutated NSCLC to anti-PD-1/PD-L1 therapy, and discusses the new strategies that may be adopted in the future.

## Introduction

Lung cancer is the leading cause of cancer-related deaths worldwide (1). Non-small cell lung cancer (NSCLC) is the most common histological subtype which constitutes more than 85% of all lung cancer cases. The prognosis of advanced NSCLC is very poor. A few subsets of NSCLC patients harboring epidermal growth factor receptor (EGFR) mutation or anaplastic lymphoma kinase (ALK) rearrangement were known to respond well to the respective molecular targeted drugs with minimum adverse reaction (2). However, targeted therapies are ineffective in most NSCLC patients whose tumors lack the oncogenic driver alterations. On the other hand, despite excellent initial response to targeted therapies, essentially all EGFR-mutant NSCLC inevitably progress over time due to acquired drug resistance (3). There is currently no further effective therapeutic options for NSCLC who develop disease progression on EGFR tyrosine kinase inhibitors (TKIs) (4).

In recent years, immunotherapy has become integrated into the treatment plan of NSCLC patients, which tremendously improved survival and quality of life in some patients (5). Anti-CTLA-4 (e.g., ipilimumab) that changed the paradigm in melanoma treatment, when tested in clinical trials did not show the expected benefit in NSCLC patients (6, 7). On the other hand, monoclonal antibodies targeting programmed death-1 (PD-1) and programmed death ligand-1 (PD-L1) have demonstrated survival benefits, long lasting responses and good safety profile over chemotherapy in patients with advanced NSCLC in several recent Phase III trials (8–11). To date, four anti-PD-1/PD-L1 monoclonal antibodies (nivolumab, pembrolizumab (anti-PD-1); atezolimumab and durvalumab (anti-PD-L1)) have been approved as 1^st^ or 2^nd^ line therapy for NSCLC patients with metastatic and locally advanced NSCLC respectively (12, 13).

PD-1 is an inhibitory receptor expressed on activated T cells, B cells and natural killer cells, which normally function to blunt the immune response. The major ligand of PD-1, PD-L1, is expressed in tumor cells and infiltrating immune cells. When PD-L1 interacts with PD-1, they suppress the T cell-mediated cancer killing effect. Anti-PD-1/PD-L1 antibodies work by binding to inhibitory PD-1 receptor on tumor-reactive T cells and PD-L1 on tumor cells, respectively. The PD-1/PD-L1 interaction is then disrupted to reactivate the anti-tumor T cell-mediated cell cytotoxicity. Clinical benefit from anti-PD-1/PD-L1 therapy is associated with high tumor mutational load, high levels of pre-treatment tumor-infiltrating T cells, and high expression of pre-treatment PD-L1 on tumor cells and tumor-infiltrating immune cells (14).

EGFR-mutant NSCLC is the most prevalent molecular subtype in lung cancer patients. However, patients with EGFR-mutant NSCLC show poor response to anti-PD-1/PD-L1 treatment. The mechanisms mediating the poor response of EGFR-mutated NSCLC patients to immunotherapy are not clear. Initial investigations revealed that EGFR-mutated NSCLC has lower PD-L1 expression and a low tumor mutational burden (TMB), thus leading to weak immunogenicity.

This review recapitulates the underlying mechanisms contributing to the inferior clinical outcomes of anti-PD-1/PD-L1 immune-checkpoint inhibitors (ICIs) in NSCLC patients bearing EGFR mutations. Novel strategies to potentiate the use of PD-1 blockade therapy in EGFR mutant NSCLC are discussed.

## Predictive Biomarkers for Selecting NSCLC Patients for Immunotherapy

Identification of predictive biomarkers to select NSCLC patients most likely responding to anti-PD-1/PD-L1 ICIs is currently an area of intensive research. The response rates of anti-PD-1/PD-L1 ICIs were estimated to be around 14-20% in unselected patients (15). The most established predictive biomarker is PD-L1 expression status of tumor cells from biopsy. It is now routinely used in clinical practice for treatment decision to select patients who may benefit the most. In fact, a number of clinical studies have reported the association between PD-L1 expression and clinical outcome in NSCLC patients [reviewed in (16)]. PD-L1 expression on tumor cells is considered not only a predictive biomarker for response to PD-1/PD-L1 ICIs but also a prognostic factor in NSCLC patients (17).

However, a recent study reported significant discrepancy in the assessment of PD-L1 tumor expression in NSCLC patients and its association with prognosis (17). Multiple PD-L1 immunohistochemical (IHC) assays with various scoring systems and cutoff values have been developed for companion diagnostic use (18, 19). Thus, appreciable differences in the correlation observed in different clinical trials may arise from the different IHC assays, the antibodies used for the assays, positivity cutoff, type of biopsies (primary versus metastasis) and staining of tumor versus immune cells (17). It will be important to standardize a universal assay to assess tumoral PD-L1 expression and also to define appropriate cut-off points (20).

On the other hand, PD-L1 is also known to be highly expressed in circulating immune cells, including dendritic cells (21) and myeloid-derived suppressor cells (22). They regulate T cell activation during antigen presentation or excessive inflammation (23, 24). It has been postulated that the baseline distribution of PD-L1 expression in systemically circulating immune cells could contribute to the therapeutic responses to PD-L1/PD-1 blockade immunotherapy. While tumoral PD-L1 expression was assessed in most clinical trials investigating anti-PD-1/PD-L1 ICIs, PD-L1 level of tumor-infiltrating immune cells was also evaluated in atezolizumab’s trials [POPLAR (25) and OAK (11)]. In both POPLAR and OAK trials, higher PD-L1 levels in both tumor cells and tumor-infiltrating immune cells were associated with improved patient survival after atezolizumab treatment. A more recent report also revealed that NSCLC patients with percentages of PD-L1+ CD11b+ myeloid cells above 30% before the start of anti-PD-L1 immunotherapy exhibited superior response rates of 50% (26). The data suggest that PD-L1 expression on myeloid cells in the systemic circulation could serve as a useful and accessible biomarker for patient stratification.

However, the utility of PD-L1 expression alone as an exclusive predictive biomarker for clinical efficacy of anti-PD-1/PD-L1 ICIs remains controversial (17). The determination of PD-L1 level alone is insufficient to understand the mechanisms of resistance to anti-PD-1/PD-L1 ICIs. It also does not explain why some PD-L1-negative patients can achieve response to treatment. PD-L2 is another ligand identified for PD-1 T cell receptor (TCR) (27). While PD-L1 is the predominant ligand for PD-1, PD-L2 could compete with PD-L1 with 2-6 fold higher affinity to PD-1 (28). However, the biological role of PD-L2 in the tumor microenvironment (TME) and as a predictive marker in NSCLC has not been definitively established. More investigations about the predictive and prognostic roles of PD-L2 are warranted.

Besides PD-L1 expression, TMB, DNA mismatch repair deficiency, extent of CD8+ cell infiltration, immune gene expression signatures and composition of the gut microbiome have also been proposed to correlate with clinical response to anti-PD-1/PD-L1 ICIs (29–32). NSCLC patients with high TMB and the smokers were found to respond better to anti-PD-1/PD-L1 ICIs (33). The potential use of TMB as a predictive marker of clinical response to anti-PD-1 therapy has been evaluated in the CheckMate026, CheckMate568 and CheckMate227 trials (34–37). NSCLC patients with high TMB showed prolonged clinical benefit and PFS to immunotherapy regardless of PD-L1 expression (38–40). It is noteworthy that lung cancer generally has higher TMB when compared with other tumor types (41). However, overall survival (OS) in anti-PD-1 ICI-treated NSCLC patients was not affected by TMB alone. Thus, further investigation about the role of TMB as a predictive biomarker is warranted before clinical implementation. Galectin-3 is a carbohydrate-binding lectin whose expression is associated with inflammatory cells including macrophage. Recently, NSCLC patients with negative or intermediate expression of galectin-3 in their tumor cells were found to demonstrate an early and durable response to pembrolizumab (42). A large multicenter clinical trial is underway to investigate the potential use of galectin-3 as a predictive marker for better patient selection for immunotherapy (42). Last but not least, NSCLC patients bearing EGFR mutations have been reported to show poor response to anti-PD-1/PD-L1 ICIs (43), which will be discussed in detail in the next section.

## NSCLC Patients Harboring EGFR Mutations Show Poor Response to Anti-PD-1/PD-L1 Immunotherapy

### Initial Enthusiasm About Using PD-1 ICIs in EGFR-Driven NSCLC According to Preclinical Studies

Early preclinical studies have reported that aberrant oncogenic EGFR signaling upregulates PD-L1 expression in NSCLC cell lines (44). PD-1 inhibitors were found to inhibit tumor cell proliferation in coculture systems of EGFR-mutant tumor and immune cells *in vitro* (44, 45). Moreover, PD-1 inhibitors were also shown to improve survival in EGFR-mutant mouse models (44). However, clinical studies have revealed an opposite result. NSCLC patients harboring EGFR mutation exhibited poorer response to PD-1/PD-L1 ICIs than those bearing wild-type EGFR (9, 11, 46, 47). More recently, a retrospective analysis conducted by Gainor et al. has revealed that EGFR mutations were associated with low clinical response to PD-1 blockade in NSCLC patients (48). The discrepancies between preclinical and clinical findings indicate a complex relationship among EGFR mutation, the immune microenvironment and therapeutic response from immunotherapy. Furthermore, EGFR TKI treatment in EGFR-driven NSCLC cell model was shown to cause PD-L1 downregulation (45), thus also deterring the utility of combining EGFR TKI with PD-1 inhibitor. In fact, the combination of EGFR TKI and PD-1 inhibitor did not lead to synergistic anticancer effect in EGFR-driven coculture system (45).

### Key Clinical Trials Evaluating Anti-PD-1/PD-L1 ICIs in EGFR-Mutant NSCLC

Advanced NSCLC patients bearing EGFR mutations only account for about 5-14% of the total number of patients recruited in the major clinical trials investigating the four approved anti-PD-1/PD-L1 ICIs (**Table 1**) (8, 9, 11, 25, 46, 51, 52). Since these clinical trials were not designed solely to investigate the role of PD-1/PD-L1 blockade immunotherapy in EGFR mutant NSCLC patients, the efficacy in EGFR mutant patients was revealed by patient subgroup analysis. CheckMate-057 is the first Phase III trial to report the clinical efficacy of PD-1/PD-L1 inhibitors in NSCLC patients bearing EGFR mutant tumors. While this trial confirmed that patients with advanced non-squamous NSCLC and progress during or after platinum-based chemotherapy survived longer with nivolumab (an anti-PD-1 monoclonal antibody) than docetaxel, subgroup analysis revealed that there was no PFS or OS benefit in patients with activating EGFR mutation (9). Patient subgroup analysis in another Phase III trial (KEYNOTE-010) evaluating pembrolizumab (another PD-1 inhibitor) also indicated that EGFR mutant NSCLC did not achieve statistically significant OS benefit from immunotherapy over salvage chemotherapy (46). In another Phase III trial (OAK) evaluating atezolizumab (an anti-PD-L1 monoclonal antibody), NSCLC patients with EGFR-mutated tumor also did not achieve OS benefit from the immunotherapy over docetaxel (11). A pooled analysis evaluating data from 3 clinical trials (CheckMate-057, KEYNOTE-010 and POPLAR) confirmed that PD-1/PD-L1 ICIs did not enhance OS versus docetaxel in advanced NSCLC patients bearing EGFR mutation (n = 186, HR = 1.05, 95% CI: 0.70-1.55, P < 0.81) (47). Furthermore, another pooled analysis which covered 5 trials (CheckMate-017, CheckMate-057, KEYNOTE-010, OAK, and POPLAR) also verified that prolonged OS was only observed in the EGFR wild-type patient group but not in the EGFR-mutant subgroup (50).

**Table 1:** Key clinical trials reporting efficacy and toxicity of PD-1/PD-L1 blockade immunotherapy in EGFR-mutant NSCLC patients.

PD-1/PD-L1 blockade therapy	Clinical trial #	Efficacy	Toxicity	Reference
Atezolizumab	OAK (Phase III; NCT02008227)	No OS benefit from atezolizumab over docetaxel in EGFR mutant versus wild-type patients (HR: EGFR mutant – 1.24 (0.71-2.18) versus EGFR wild-type – 0.69 (0.57-0.83)	Grade 3-4 treatment-related adverse events: 15% with atezolizumab group versus 43% with docetaxel group	(11)
Nivolumab	CheckMate 057 (Phase III; NCT01673867)	Median OS was 12.2 months (n=292) in nivolumab group versus 9.4 months in docetaxel group (n=290). However, subgroup analysis in EGFR mutated patients did not show PFS or OS benefit from nivolumab (HR=1.18 (0.69-2.00)).	Grade 3-5 treatment related adverse events were reported in 10% of nivolumab and 54% of docetaxel-treated patients	(9)
Pembrolizumab	KEYNOTE-010 (Phase III; NCT01905657)	No OS benefit from pembrolizumab over docetaxel in EGFR mutant versus wild-type patients (HR: EGFR mutant – 0.88 (0.45-1.70) versus EGFR wild-type 0.66 (0.55-0.80))	Grade 3-5 treatment-related adverse events: 13% with pembrolizumab group versus 35% with docetaxel group	(46)
Pembrolizumab	Phase II; NCT0287994	The efficacy of pembrolizumab was evaluated in TKI-naïve NSCLC patients with EGFR mutation and PD-L1 positive tumors. None of the patients with EGR-mutant NSCLC responded. Enrollment was ceased due to lack of efficacy after 11 of the 25 planned patients were treated.	--	(49)
Nivolumab, Pembrolizumab, Atezolizumab	Pooled analysis (CheckMate 057, KEYNOTE 010 and POPLAR)	PD-1/PD-L1 blockade immunotherapy did not enhance OS versus docetaxel in advanced NSCLC patients bearing EGFR mutation (n=186, HR=1.05, 95% CI: 0.70-1.55, P<0.81)	--	(47)
Nivolumab, pembrolizumab, atezolizumab	Pooled analysis (CheckMate 017, 057, 063, 003)	PD-1/PD-L1 blockade immunotherapy prolonged OS in EGFR wild-type subgroup (HR=0.67; 95% CI: 0.60=0.75; P<0.001) but not in EGFR mutant subgroup (HR=1.11; 95% CI: 0.80-1.53; P=0.54)	--	(50)

CI, confidence interval; HR, hazard ratio; OS, overall survival; PD-1, Programmed death-1; PD-L1, Programmed death ligand-1.

A Phase II clinical trial (NCT0287994) was conducted to specifically evaluate the efficacy of pembrolizumab (anti-PD-1 monoclonal antibody) in TKI-naïve EGFR-mutant advanced NSCLC patients whose tumors have high PD-L1 expression (49). The trial enrolment was halted due to lack of efficacy after 11 of the 25 planned patients received the immunotherapy. Thus, the patient number is very small because of premature closure of the trial, none of the patients bearing EGFR mutations responded to pembrolizumab (49). Based on these clinical findings, the National Comprehensive Cancer Network (NCCN) clinical practice guidelines of NSCLC (version 4, 2021) did not recommend immunotherapy for the treatment of EGFR-mutant NSCLC patients.

### Efficacy of Anti-PD-1/PD-L1 ICIs in NSCLC Bearing the Two Most Common EGFR Sensitizing Mutation Subtypes

Interestingly, the heterogeneity of EGFR mutation subtypes was found to cause variations in the therapeutic efficacy of anti-PD-1/PD-L1 ICIs. A multicenter retrospective study analyzed the clinical data of 171 EGFR mutant NSCLC patients on treatment with PD-1/PD-L1 ICI alone or in combination with CTLA4 inhibitor (53). While patients harboring EGFR exon 19 deletion or L858R mutation shown less benefit from immunotherapy than the EGFR wild-type group, the L858R group exhibited more favorable response than the exon 19 deletion group (ORR, 7% in EGFR 19 deletion subgroup versus 16% in L858R subgroup versus 22% in wild-type subgroup). However, EGFR T790M status and PD-L1 expression did not affect response and survival outcomes to anti-PD-1/PD-L1 ICIs. NSCLC tumors bearing EGFR exon 19 deletion was found to have a lower tumor mutation burden compared with the EGFR L858R subtype despite similar smoking history. Therefore, screening for EGFR mutation subtypes could be useful for personalized use of PD-1/PD-L1 ICIs in EGFR-mutant NSCLC patients. Further studies with larger patient cohorts are warranted.

### Mechanisms Contributing to the Poor Efficacy of Anti-PD-1/PD-L1 Therapy in EGFR Mutant NSCLC

#### PD-L1 Expression

PD-L1 expression in tumor tissues is the most extensively studied predictive biomarker for clinical response to PD-1/PD-L1 inhibitors. Status of tumoral PD-L1 expression is now routinely used for selecting patients who could benefit the most. The contribution of PD-L1 expression to poor efficacy of anti-PD-1/PD-L1 therapy in EGFR mutant NSCLC is controversial. A number of studies have reported that high PD-L1 expression is found more frequently in EGFR-mutant than EGFR-wild type lung tumor tissues (44, 54–56). The activation of the PD-1 pathway is thus believed to contribute to immune escape in EGFR-driven NSCLC. In contrast, another study has found a decreased PD-L1 expression in tumor tissues from NSCLC patients bearing EGFR mutation (57). More recently, a pooled analysis of 15 clinical studies also revealed that patients with EGFR mutation have decreased PD-L1 expression (58). Analysis of The Cancer Genome Atlas (TCGA) and the Guangdong Lung Cancer Institute (GCLI) cohort have also confirmed an inverse correlation between EGFR mutation and PD-L1 expression in tumor tissues (58). On the other hand, other studies have reported a lack of correlation between the expression of PD-L1 and PD-L2 in patients with different EGFR mutation status (59). With these conflicting findings, the use of PD-L1 expression alone could not adequately predict and explain why EGFR-mutant NSCLC exhibits poor response to PD-1/PD-L1 inhibitors.

#### Tumor Mutational Burden (TMB)

In a recent study investigating the impact of TMB on clinical outcomes of NSCLC patients treated with EGFR TKIs, TMB was found to be remarkably lower in EGFR-mutated tumors (n = 153) than EGFR wild-type tumors (n = 1,849) (median 3.77 versus 6.12 mutations/Mb; *P* < 0.0001) (60). To this end, the association of higher TMB with tobacco smoking leading to better outcomes with PD-1/PD-L1 ICIs is well documented (9, 11, 34, 37, 46, 61). A recent meta-analysis also revealed that never smokers are less responsive to PD-1/PD-L1 inhibitors (62). Interestingly, EGFR mutant NSCLC is more enriched in the never smoking population (63).

Among the more common sensitizing EGFR mutations, TMB in the exon 19 deletion cohort was found to be lower than that in the L858R cohort (60). Hastings et al. has recently reported that clinical outcomes (OS and ORR) with PD-1 ICIs were worse in patients with exon 19 deletion than patients harboring L858R mutation (53). PD-L1 and smoking status were similar in the two patient subpopulations. Further TMB analyses of the two patient cohorts suggested that the higher TMB in EGFR L858R mutation could contribute to the differential responses to PD-1 ICIs.

In fact, specific subset of EGFR-mutant NSCLC patients have been shown to preferentially benefit from PD-1/PD-L1 ICIs to some extent (64–68). In a retrospective study evaluating NSCLC patients from the IMMUNOTARGET registry, the response of 125 pre-treated EGFR-mutated patients with ICI monotherapy was compared among patients with different EGFR mutation subgroups (69). The ORR was not notably affected by PD-L1 expression levels, smoking status or previous lines of treatment, but was significantly different in the various EGFR mutation subgroups (3.7%, 9.5%, 20.8% and 11.8%, respectively, in EGFR T790M, exon 19 deletion, L858R, and other EGFR mutation subgroups) (69). This is in line with the aforementioned findings of Hastings et al., demonstrating more favorable outcomes of L858R (coincident with higher TMB) than exon 19 deletion (coincident with lower TMB). The combination of nivolumab and erlotinib in 21 EGFR mutated NSCLC patients has been evaluated by Gettinger et al. (64). Intriguingly, patients bearing the EGFR L858R mutation were achieving longer survival benefit than those harboring other EGFR mutations (64). While TMB in the tumor tissues was not assessed in the study, the findings are consistent with the fact that TMB in EGFR L858R mutated NSCLC favors better response to PD-1 ICIs.

#### Immunosuppressive Tumor Microenvironment (TME) in EGFR-Mutant Tumors

**Figure 1:**
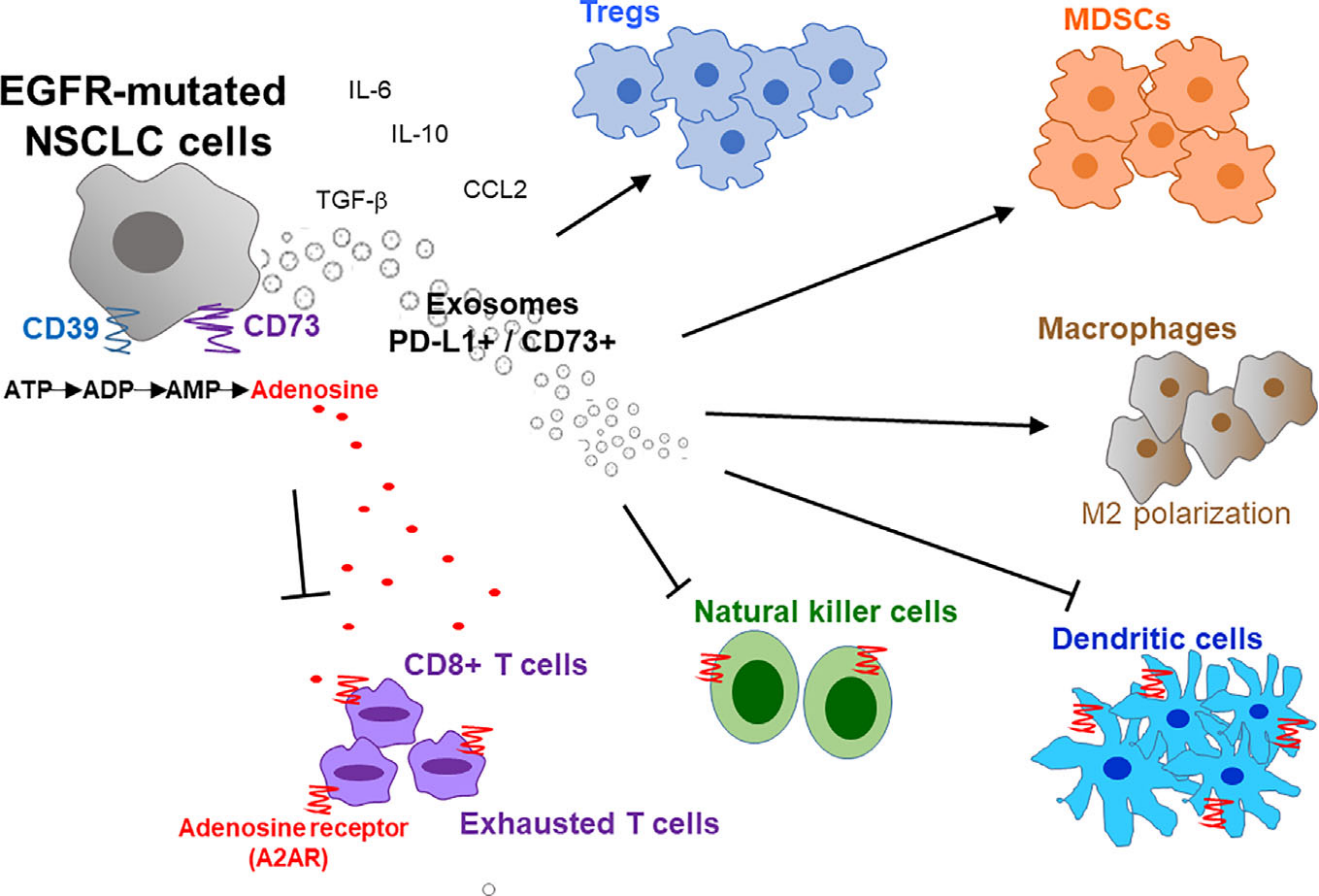
Immunosuppressive tumor microenvironment (TME) in EGFR-mutated NSCLC. EGFR mutations promote an immunosuppressive TME by interfering with several intracellular pathways and modulating immune accessory cells including tumor-infiltrating lymphocytes (TILs), natural killer cells (NK), T-regulatory cells (Tregs), myeloid-derived suppressor cells (MDSCs) and tumor-associated macrophages (TAMs). Overexpression of CD39/CD73 in EGFR-mutated NSCLC induces high extracellular production and release of adenosine that inhibit the activity of innate and adaptive immune system cells and endothelial cells in TME. Activation of CD39 triggers the de-phosphorylation of ATP to ADP, and subsequently to AMP. On the other hand, CD73 catalyzes the hydrolysis of AMP to adenosine and phosphate. The increased level of extracellular adenosine bind to A2A adenosine receptor (A2AR) expressed by both adaptive and innate immunity, thereby inhibiting the activity of various immune cells. Moreover, exosomes secreted from EGFR-mutated NSCLC cells also increase PD-L1+/CD73+ expression and extracellular adenosine release to promote immunosuppression. IL, interleukin; M2, macrophages 2; ATP, adenosine triphosphate; ADP, adenosine diphosphate; AMP, adenosine monophosphate; CCL2, C-C motif chemokine ligand 2.

A typical tumor mass consists of not only a heterogeneous population of cancer cells but also a variety of neighboring host cells, tumor-infiltrating lymphocytes, extracellular matrix proteins, and other secreted factors, which collectively referred to as the TME. Tumors have been classified into 4 different TME types according to the presence or absence of tumor-infiltrating lymphocytes (TIL) and PD-L1 expression (Type I: TIL^+^, PD-L1^+^; Type II: TIL^-^, PD-L1^-^; Type III: TIL^-^, PD-L1^+^; and Type IV: TIL^+^, PD-L1^-^) (70). Type I tumors were found to the only subtype that responds to PD-1/PD-L1 inhibitor (70). Therefore, besides PD-L1 expression, high level of TIL is critical to allow PD-1/PD-L1 inhibitors to work. Similarly, Chen et al. divided tumors into different immunity phenotypes (immune-desert phenotype; immune-excluded phenotype; and inflamed phenotype) according to a set of tumor, host, and environment factors (71). Using Chen’s classification, tumors with the immune-desert and immune-excluded phenotypes are resistant to PD-1/PD-L1 inhibitors (71). Importantly, there is a close correlation between EGFR mutations and an uninflamed TME with immunological tolerance and weak immunogenicity (48, 58). This correlation may explain the inferior response of EGFR-mutant NSCLC to PD-1 blockade therapy. The overexpression of CD73 has also been reported in EGFR mutant NSCLC, thus resulting an immunosuppressive TME and reduced IFN gamma signature (72). **Figure 1** depicts the characteristic composition and function of the immunosuppressive TME in EGFR-mutated NSCLC cells.

EGFR-mutant NSCLC is characterized by its aberrant activation of the EGFR signaling pathway. To this end, activation of EGFR signaling has been reported in numerous studies to participate in immunosuppression and immune escape. Regulatory T cells (Tregs) play a critical role in suppressing the immune response to self and foreign particles, which help prevent autoimmune disease. They generally suppress the induction and proliferation of effector T cells. Amphiregulin (AREG) is an EGF-like growth factor and it is frequently upregulated in tumors (73). AREG is a known ligand of EGFR (74). Importantly, AREG is critical for Treg function *in vivo*, thus providing a mechanistic link between the EGFR signaling and regulation of the immune system (75). Wang et al. reported that AREG maintains the suppressive function of Tregs *via* the EGFR/GSK-3β/Foxp3 machinery *in vitro* and *in vivo*, thus confirming the importance of EGFR signaling in the regulation of Tregs (76). Recently, a long noncoding RNA lnc-EGFR has been shown to stimulate Treg differentiation and promote immune invasion of hepatocellular carcinoma *via* an EGFR-dependent signaling pathway (77). Moreover, the inhibition of EGFR signaling by gefitinib has been shown to alter the immune environment of the targeted cancer *in vitro* and *in vivo*, probably by reducing the number of Tregs in the tumors (78).

Myeloid-derived suppressor cells (MDSCs) are immature myeloid cells that suppress immune responses. MDSCs expand during cancer, infection and inflammatory diseases. A recent study reported that EGFR TKI therapy alters the TME in EGFR mutant NSCLC and elevates the level of mononuclear MDSCs (79). The serum level of inflammatory factors IL-10 and CCL-2 was also found to be increased *in vivo* after EGFR TKI treatment (79). The increase in MDSC and inflammatory factors associated with EGFR TKI treatment has been proposed to explain why most EGFR TKI-resistant NSCLC patients are also refractory to anti-PD-1/PD-L1 ICIs (79). Moreover, MDSCs are known to inhibit IL-2 and anti-CD3/CD28 mAb-induced T cell amplification and Th1 polarization but induce apoptosis in T cells in an IDO-dependent manner (80). To this end, the activation of STAT3 (an important downstream signaling molecule of the EGFR pathway) is required for IDO expression (80). Therefore, STAT3 activation is essential for immune suppression of MDSCs. In fact, persistent activation of STAT3 has been shown to promote MDSC-mediated immune suppression in lung cancer (81).

#### Yes-Associated Protein (YAP)

YAP is a major mediator of the Hippo pathway and it has been shown to promote cancer progression, drug resistance and metastasis in NSCLC (82). Accumulating evidence suggests that YAP also plays critical role in cancer immunity. YAP interacts with interferon regulatory factor 3 to negatively regulate innate immunity (83). YAP has also been reported to regulate tumor–associated immune cells (including MDSCs, macrophages, and Tregs) in the TME (83–85). In NSCLC tumor specimens, high nuclear YAP staining is associated with positive PD-L1 expression (86). Genetic knockdown or chemical inhibition of YAP was shown to reduce mRNA and protein expression of PD-L1 in NSCLC cell lines (86). On the other hand, forced expression of YAP was shown to increase PD-L1 protein expression in NSCLC A549 cells (87). Recently, a gefitinib-resistant PC9 cell line has been shown to express higher protein level of YAP and PD-L1 than the parental cells (88). Importantly, YAP knockdown could reduce PD-L1 expression in gefitinib-resistant PC9 cells (88). It is also noteworthy that the EGFR signaling pathway has crosstalk with the Hippo/YAP pathway, which positively regulating the YAP oncogenic function in various cancers including NSCLC (89).

### Preferential Response of NSCLC Patients With Uncommon EGFR Mutations to PD-1 Blockade Therapy

Emerging evidence from a few recent studies suggest that the efficacy of PD-1 blockade therapy is relatively more favorable in NSCLC patients bearing uncommon EGFR mutations compared to those with the classical mutations (46, 69, 90). Approximately 10% of EGFR mutant NSCLC is classified as the uncommon subtypes (91), including G719X, L861Q, S768I, and exon 20 insertion, which have different clinicopathological characteristics and response to EGFR TKIs (92–96). Most recently, Chen et al. investigated the clinical response of Chinese NSCLC patients harboring uncommon EGFR mutations to PD-1/PD-L1 inhibitors and the underlying mechanisms (90). They tied the favorable response of the NSCLC patients with uncommon EGFR mutations to the high incidence of concomitant PD-L1 expression and CD8+ tumor-infiltrating lymphocytes within TME (90). In a retrospective efficacy analysis of PD-1 inhibitors conducted by a Japanese group, NSCLC patients harboring uncommon EGFR mutations and without T790M mutations were associated with significantly longer PFS than those with common EGFR mutations or with T790M mutation (97). This retrospective study was limited by the small sample size (n = 27). Further investigations are warranted to identify the clinical biomarkers useful for predicting the ICI responders with EGFR mutations.

## Toxicity Experienced on Combination Treatment With EGFR TKI and Immunotherapy

Immunotherapy generally has a lower incidence of adverse reactions than chemotherapy. However, ICIs are known to mediate inflammatory side effects commonly referred to as immune-related adverse events (irAEs) (98). The etiology leading to irAEs is largely unknown but it is believed to be caused by the disruption of immunologic homeostasis (99). The occurrence of irAEs generally predicts treatment efficacy of ICIs in NSCLC (100, 101), and it also triggers treatment discontinuation and premature termination of clinical trials. A Phase 1b TATTON trial (NCT02143466) investigating osimertinib (3^rd^ generation EGFR TKI) plus nivolumab was stopped early because of the high incidence (38%) of interstitial lung disease (102). Another Phase III open-label CAURAL trial (NCT02454933) evaluating the combination of osimertinib and durvalumab in EGFR T790M positive NSCLC patients was also prematurely terminated due to safety concerns (103). irAEs can affect one or multiple organ systems. The incidence of grade 3 or above toxicities is around 7-13% in NSCLC patients treated with anti-PD-1/PD-L1 ICIs (104). It is noteworthy that toxicity experienced upon combination treatment with immunotherapy and EGFR TKIs can be severe, difficult to predict and with unusual forms of presentation. Specific laboratory tests and regular physical examinations should be conducted to facilitate early detection of irAEs and their effective management (105, 106).

## Hyperprogressive Disease (HPD) Associated With PD-1/PD-L1 Blockade in NSCLC Patients

Several recent studies have reported a paradoxical deleterious effect of anti-PD-1/PD-L1 immunotherapy, which is described as “hyperprogressive disease (HPD)”, in a subset of patients (106, 107). HPD is characterized as an unexpected and fast progression in tumor volume and rate, poor survival of patients and early fatality (108).

HPD has been defined in different studies using 5 different criteria (109). Various parameters including tumor growth rate, tumor growth kinetics and time to treatment failure were used to define and quantify the incidence of HPD (110). In a recent retrospective cohort study of NSCLC patients, these 5 definitions of HPD were found to be associated with different tumoral behaviors. Kas et al. proposed a new definition of HPD, which is based on ΔTGR (tumor growth rate) greater than 100 (110). This new definition appeared to be more closely associated with the expected characteristics for HPD (i.e., rapid increase in tumor kinetics and poor patient survival). Numerous biomarkers associated with HPD have been proposed, which may be used to stratify patients for anti-PD-1/PD-L1 immunotherapy. Among them, EGFR mutation represents an important tumor cell biomarker linked with HPD after immunotherapy (108). HPD has been reported in 20% of patients with EGFR mutations and it was associated with worse clinical outcome. EGFR mutations were known to upregulate cell surface inhibitory receptors (e.g., PD-1/PD-L1 and CTLA-4), cytokines and immunosuppressive cells, subsequently driving innate immune resistance (44). The precise role of EGFR mutations in HPD warrants further investigation.

## Novel Strategies to Potentiate PD-1/PD-L1 Blockade Immunotherapy in EGFR Mutant NSCLC

A promising therapeutic approach is to combine a PD-1/PD-L1 ICI with chemotherapy, EGFR TKI, or other type of ICI with an aim to increase the immunogenicity of tumor cells, or to inhibit immunosuppressive signaling in the TME (111).

### Combination of PD-1/PD-L1 Blockade Therapy and Conventional Chemotherapy

In lung cancer patients without targetable mutations, the addition of PD-1/PD-L1 blockade therapy to standard chemotherapy has been shown to give rise to significantly longer OS and PFS than chemotherapy alone (10). Therefore, it is also speculated that addition of PD-1/PD-L1 immunotherapy to chemotherapy in NSCLC patients with EGFR mutation could also achieve desirable clinical outcomes.

Antitumor effect from conventional chemotherapy is not solely attributed to the direct tumor cell cytotoxicity, but it is also mediated by the restoration of immunosurveillance. To this end, antitumor immune response and re-establishment of immunosurveillance can be primed by immunogenic cell death (ICD). ICD comprises the release of damage-associated molecular patterns (DAMPs) from dying tumor cells that result in activation of tumor-specific immune responses (112). This can trigger long-term efficacy of anticancer drugs by combining direct cancer cell killing and antitumor immunity. ICD-induced DAMPs include surface-exposed calreticulin (CALR) and secreted ATP, annexin A1, type I interferon, and high mobility group box 1 (113). A number of classical chemotherapeutic drugs, including anthracyclines, cyclophosphamide, oxaliplatin, and paclitaxel, are known to elicit ICD (114). In mice bearing KRAS-positive and TP53-negative NSCLC tumor xenograft, two immunogenic chemotherapeutic drugs (oxaliplatin and cyclophosphamide) were shown to strongly enhance T cell infiltration of the tumors and sensitize them to subsequent checkpoint inhibition targeting both CTLA-4 and PD-1 (115).

A few recent clinical reports have corrobated the hypothesis that pretreatment with ICD-inducing anthracyclines or irradiation could potentiate the efficacy of ICIs in various tumor types including NSCLC (116) (**Table 2**). The PACIFIC study is a Phase III trial that evaluated durvalumab (PD-L1 antibody) as consolidation therapy in stage III NSCLC patients (51). The enrolled subjects did not present disease progression after 2 or more cycles of platinum-based chemotherapy (51). The study showed a significantly longer median PFS in the durvalumab cohort (16.8 months) than that in the placebo cohort (5.6 months). Importantly, in subgroup analysis, patients with EGFR-mutations demonstrated slightly more clinical benefit from durvalumab after chemoradiotherapy (platinum doublet chemotherapy administered with definitive-dose radiotherapy) (51). An open-label Phase III trial (Checkmate 722; NCT02864251) has also been conducted to compare the efficacy of nivolumab plus chemotherapy and nivolumab plus ipilimumab with chemotherapy alone (117). The Checkmate 722 study enrolled patients with EGFR-mutated metastatic or recurrent NSCLC who progressed on 1^st^ or 2^nd^ line EGFR TKIs (117). The final result has not been reported yet. Another ongoing Phase III trial (KEYNOTE-789; NCT03515837) is evaluating the efficacy of pembrolizumab in combination with chemotherapy (pemetrexed, carboplatin or cisplatin). Unlike the CheckMate 722 trial, KEYNOTE-789 will also recruit NSCLC patients bearing EGFR T790M who have acquired resistance to osimertinib (118).

**Table 2:** Representative clinical trials evaluating the combination of PD-1/PD-L1 blockade immunotherapy and conventional chemotherapy in EGFR-mutant NSCLC patients.

Clinical trial #	PD-1/PD-L1 blockade therapy	Chemotherapy	Key findings	Reference
PACIFIC	Durvalumab (PD-L1 antibody)	Platinum-based chemotherapy	- Phase III trial evaluating durvalumab as consolidation therapy in stage III NSCLC patients who did not present disease progression after 2 or more cycles of chemotherapy. - In patient subgroup analysis, patients with EGFR mutations demonstrated slightly more clinical benefit from durvalumab after chemoradiotherapy.	(51)
Checkmate 722 (NCT02864251)	Nivolumab (PD-1 antibody)	Pemetrexed, cisplatin, or carboplatin	- Open-label phase III trial enrolling ~500 patients with confirmed stage IV or recurrent EGFR mutated NSCLC progressed on prior EGFR TKI therapy - Efficacy of nivolumab plus chemotherapy, nivolumab plus ipilimumab and chemotherapy alone was compared. - Final result has not been reported.	(117)
KEYNOTE-789 (NCT03515837)	Pembrolizumab (PD-1 antibody)	Pemetrexed, carboplatin or cisplatin	- Ongoing Phase II trial which compared efficacy of pembrolizumab and its combination with chemotherapy - It also recruited NSCLC patients bearing EGFR T790M who have acquired resistance to osimertinib	(118)

### Combination of PD-1/PD-L1 Immunotherapy With Targeted Therapy

High levels of intra-tumoral T cells and tumor antigenicity have been shown to correlate with favorable response from immunotherapy (119). Thus, therapies capable of increasing these factors may be combined effectively with ICIs to enhance the treatment outcome. In addition to the specific effects on oncogenic signaling pathways, a few targeted therapeutic agents are also known to increase tumor antigen presentation (120, 121), promote intra-tumoral T cell infiltration (122), or upregulate PD-1/PD-L1 expression (123). Therefore, it is logical to combine these targeted therapies with immunotherapies.

#### EGFR TKIs

EGFR TKIs have been reported to cause immunogenic apoptosis of tumor cells (124), and subsequently releasing aberrant intracellular antigens and recruiting T cells *via* interferon-γ-induced major histocompatibility complex class I presentation (120). A few clinical studies have been conducted to evaluate the combination of PD-1-based immunotherapy and EGFR targeted therapy in EGFR TKI-naïve and/or pretreated EGFR-mutant NSCLC patients (**Table 3**).

**Table 3:** Representative clinical trials evaluating the combination of PD-1/PD-L1 blockade immunotherapy and targeted therapy in EGFR-mutant NSCLC patients.

Clinical trial #	PD-1/PD-L1 blockade therapy	Targeted therapy	Key findings	Reference
CheckMate 012 (NCT01454102)	Nivolumab	Erlotinib (EGFR TKI)	- Phase I trial evaluating combination of nivoluman and various other agents including erlotinib - At least 4 out of the 20 recruited NSCLC patients with acquired resistance to EGFR TKI achieved clear benefit from combination of nivolumab and erlotinib (ORR = 15%, including 1 CR)	(125)
NCT02013219	Atezolizumab	Erlotinib (EGFR TKI)	- Phase I trial in EGFR TKI-naïve and –treated NSCLC patients - Combination of atezolizumab and erlotinib was well tolerated and it exhibited favorable efficacy compared with prior reports of erlotinib monotherapy. ORR = 75% and median PFS = 15.4 months	(126)
KEYNOTE-021 (NCT02039674)	Pembrolizumab	Gefitinib or erlotinib (EGFR TKI)	- Phase I/II trial evaluating the combination of pembrolizumab with erlotinib or gefitinib in advanced NSCLC patients bearing EGFR mutation - Pembrolizumab plus erlotinib did not improve ORR compared with previous EGFR TKI monotherapy - Pembrolizumab plus gefitinib combination caused grade 3/4 liver toxicity in 5 out of 7 patients, resulting in premature treatment discontinuation	(126)
NCT02088112	Durvalumab	Gefitinib (EGFR TKI)	- Open-label multicenter Phase I trial evaluating combination of gefitinib and durvalumab in patients with EGFR-mutant and EGFR TKI-naïve NSCLC - No significant improvement in PFS or ORR compared with gefitinib monotherapy previously reported in similar patient populations. - Gefitinib-naïve patients: ORR=63.6%; DoR=9.2 months; PFS=10.1 months - Gefitinib-pretreated patients: ORR=70.0%; DoR=12.6 months; PFS=12.0 months	(127)
TATTON (NCT02143466)	Durvalumab	Osimertinib (EGFR TKI)	- Phase Ib trial investigating the safety and tolerability of osimertinib and durvalumab combination - 38% of subjects developed serious interstitial pneumonitis	(102)
IMpower150 (NCT02366143)	Atezolizumab	Bevacizumab (anti-VEGF monoclonal antibody)	- Open-label Phase III study comparing atezolizumab + chemotherapy + bevacizumab (ABCP group) versus chemotherapy + bevacizumab (BCP group) in metastatic and chemotherapy-naïve NSCLC patients - ABCP group achieved significantly longer PFS (8.3 versus 6.8 months) and OS (19.2 versus 14.7 months) than BCP group, regardless of PD-L1 expression and EGFR/ALK genetic alteration status	(128)

CR, complete response; DoR, duration of response; ORR, objective response rate; OS, overall survival; PFS, progression free survival.

CheckMate 012 (NCT01454102) is a multi-arm Phase 1 study evaluating the combination of nivolumab and different agents including erlotinib in advanced NSCLC with small sample size (n = 20) (125). At least 4 out of the 20 patients with acquired resistance to EGFR TKI were shown to achieve clear benefit from the addition of nivolumab to EGFR TKI therapy [objective response rate (ORR) = 15%, including 1 complete response (CR)] (125). The combination of atezolizumab and erlotinib has been evaluated in another Phase I clinical trial in EGFR TKI-naïve and –treated NSCLC patients (NCT02013219; n = 28) (126). Atezolizumab plus erlotinib was shown to demonstrate a tolerable safety profile and favorable efficacy compared with prior reports of erlotinib monotherapy. ORR was 75% and median PFS was 15.4 months (126).

On the other hand, the combination of EGFR TKIs with PD-1/PD-L1 inhibitors did not demonstrate favorable clinical efficacy in EGFR-mutated NSCLC patients in a few other trials. In the Phase I/II KEYNOTE-021 trial (NCT02039674), the combination of erlotinib (n = 12) or gefitinib (n = 7) with pembrolizumab was evaluated in EGFR-mutant advanced NSCLC patients (129). While pembrolizumab plus erlotinib produced similar adverse events as erlotinib monotherapy, pembrolizumab plus gefitinib combination caused grade 3/4 liver toxicity in five out of seven patients and resulting in premature treatment discontinuation. Disappointingly, pembrolizumab plus erlotinib did not improve ORR compared with previous EGFR TKI monotherapy studies (129). Another open-label multicenter Phase I trial (NCT02088112) has also been recently conducted to evaluate the combination of gefitinib and duvalumab in patients with EGFR-mutant and EGFR TKI-naïve NSCLC (127). Durvalumab and gefitinib in combination was shown to produce higher toxicity than either drug alone. There was no significant improvement in PFS or ORR compared with gefitinib monotherapy previously reported in similar patient populations. To the best of our knowledge, no other Phase III trials investigating the combination EGFR TKI and PD-1 inhibitors in EGFR TKI-naïve patients are currently planned or actively recruiting.

In preclinical studies, the 3^rd^ generation EGFR TKI, osimertinib, has been shown to enhance the antitumor efficacy of PD-1/PD-L1 blockade therapy by increasing CD8+ T cell infiltration in tumors (130). However, in a Phase Ib trial TATTON (NCT02143466) investigating the safety and tolerability of combining osimertinib and durvalumab, 38% of patients developed serious interstitial pneumonitis and thus the poor safety profile renders the combination not feasible (102). In an animal study using EGFR mutated tumor-bearing mouse model, osimertinib (but not gefitinib) combined with anti-PD-L1 therapy was shown to cause pneumonitis and injury to lung tissues (124).

#### Vascular Endothelial Growth Factor Receptor (VEGFR) TKI

IMpower150 was an open-label Phase III study (NCT02366143) comparing atezolizumab ± chemotherapy + bevacizumab (ABCP group) versus chemotherapy + bevacizumab (BCP group) in metastatic NSCLC patients who had not previously received chemotherapy (128). Bevacizumab is an anti-VEGF monoclonal antibody and its combination with chemotherapy has been approved for the treatment of metastatic NSCLC (131). Apart from the well-known antiangiogenic effects of bevacizumab (132), the inhibition of VEGF has also been shown to mediate immunomodulatory effects (14, 133, 134). Thus, the efficacy of atezolizumab may be enhanced by the addition of bevacizumab to reverse VEGF-mediated immunosuppression (134, 135). Encouragingly, the ABCP group was shown to achieve significantly longer PFS (8.3 months versus 6.8 months) and OS (19.2 months versus 14.7 months) than the BCP group, regardless of PD-L1 expression and EGFR/ALK genetic alteration status (128) (**Table 3**).

### Combination of PD-1/PD-L1 ICIs With Other Immunotherapies

Both CTLA-4 and PD-1 ICIs demonstrated impressive durable antitumor response and they had a manageable safety profile. However, benefits of monotherapy were limited by low response rates and only a fraction of patients were found to be responsive (136). Combination of CTLA-4 and PD-1/PD-L1 blockade was proposed to increase the response rates and patient survival rates. It was thought that blockade of CTLA-4 (primarily involved in regulation of T cell activation in lymph nodes and in suppression of DC activity *via* Treg cells) could act synergistically with blockade of PD-1 (mainly involved in inhibition of effect T cells and NK cell activation in peripheral tissues and in induction of Treg cell differentiation) (137).

Multiple studies have investigated the combination of PD-1/PD-L1 plus CTLA-4 antibodies in treating NSCLC (**Table 4**). The first study is a phase Ib trial that evaluated the safety and efficacy of durvalumab (anti-PD-L1) and tremelimumab (anti-CTLA-4) combination. Encouraging clinical activity was observed in NSCLC patients with PD-L1 positive as well as PD-L1 negative tumors, with investigator assessed confirmed ORR in 23% patients (138). Importantly, this study revealed that the antitumor effect of the combination does not depend on PD-L1 expression. Thus, this might provide a new treatment option for patients with negative PD-L1 expression (138). In a phase III trial evaluating chemotherapy-naïve stage IV or recurrent NSCLC patients, the combination of nivolumab and ipilimumab achieved ORR of 45.3%, 1-year progressive free survival rate of 42.6% and median PFS of 7.2 months (139). The relative incidence of disease progression or death was significantly lower in nivolumab plus ipilimumab combination compared to chemotherapy alone group (HR for disease progression or death, 0.58, p < 0.001). In another phase II study, the efficacy and safety of nivolumab plus “low dose” ipilimumab as first line treatment for metastatic NSCLC was investigated (140). The association of efficacy with PD-L1 expression and TMB was also assessed. The ORR achieved by the combination was found to be higher in patients with TMB of at least 10 mutations per megabase and it was not dependent on PD-L1 expression (140).

**Table 4:** Representative clinical trials investigating the combination of PD-1/PD-L1 and CTLA-4 blockade immunotherapies in NSCLC.

Clinical trial #	PD-1/PD-L1 inhibitor	CTLA-4 inhibitor	Key findings	Reference
NCT02000947 (Phase Ib)	MEDI4736 (anti-PD-L1 mAb)	Tremelimumab	- Advanced NSCLC patients - ORR, 23% - Grade 3-4 AEs, 35%	(138)
NCT01454102 (Phase I)	Nivolumab	Ipilimumab	- Untreated advanced NSCLC - ORR, 47% - Median PFS, 8.1 months - 24-week PFS rate, 68% - Grade 3-4 AEs, 37%	(139)
NCT02659059 (Phase II)	Nivolumab	Ipilimumab	- Untreated advanced (Stage IV) NSCLC patients - In patients with TMB > 10 mutations/megabase: ORR, 44% Median PFS, 7.1 months 6-month PFS rate, 55% Grade 3-4 AEs, 29%	(140)
NCT02477826 (Phase III)	Nivolumab	Ipilimumab	- Untreated advanced (Stage IV) NSCLC patients - In patients with TMB > 10 mutations/megabase: ORR, 45% Median PFS, 7.2 months 12-month PFS rate, 43% HR for disease progression or death, 0.58 Grade 3-4 AEs, 31%	(139)

AE, adverse event; HR, hazard ratio; mAb, monoclonal antibody; ORR, objective response rate; PFS, progression free survival; TMB, tumor mutational burden.

### Combination of PD-1/PD-L1 Blockade Immunotherapy With Other Miscellaneous Therapies

Interleukin-10 (IL-10) is known to possess anti-inflammatory and CD8+ T cell stimulating activities (141). Pegilodecakin (pegylated IL-10) is a first-in-class long-acting IL-10 receptor agonist that induces oligoclonal T cell expansion (142). It has demonstrated single-agent activity in advanced solid tumors (143) (**Table 5**). IVY is a multicenter, multicohort, open-label, phase Ib trial (NCT02009449) evaluating the combination of pegilodecakin and pembrolizumab or nivolumab for patients with advanced solid tumors (144). The combination of pegilodecakin with anti-PD-1 monoclonal antibodies demonstrated a manageable toxicity profile and promising antitumor activity. Th ORR was relatively higher for NSCLC (43%), than that in renal cell carcinoma (40%) and melanoma (10%) (144). The favorable responses were also observed when PD-1/PD-L1 blockade immunotherapy only produced limited benefit, such as low PD-L1 expression, low TMB and liver metastasis. Since subgroup analysis focusing on EGFR-mutant patients were not available from the study, further investigation is needed to verify its clinical usefulness for NSCLC patients with EGFR mutations.

**Table 5:** Representative studies (clinical trials and animal studies) evaluating the combination of PD-1/PD-L1 blockade immunotherapy and other miscellaneous therapies in EGFR-mutant NSCLC.

Type of study	PD-1/PD-L1 blockade therapy	Other miscellaneous therapy	Key findings	Reference
Clinical trial: IVY (NCT02009449)	Pembrolizumab or nivolumab	Pegilodecakin (PEGylated IL-10) (first-in-class long-acting IL-10 receptor agonist that induces oligoclonal T cell expansion)	- Multicenter, multicohort, open-label, phase Ib trial evaluating the drug combination in patients with advanced solid tumors (including NSCLC, renal cell carcinoma, and melanoma) - ORR for the drug combination was higher for NSCLC (43%) than that in renal cell carcinoma (40%) and melanoma (10%) - However, patient subgroup analysis focusing on EGFR-mutant patients were not available from the study	(144)
Clinical trial: TACTI-002 (NCT03625323)	Pembrolizumab	IMP321 (recombinant LAG-3Ig fusion protein)	- Ongoing Phase II trial investigating the combination in patients with previously untreated unresectable or metastatic NSCLC, recurrent PD-X refractory NSCLC, or metastatic HNSCC - Pilot results demonstrated that combination achieved an ORR of 47% in advanced NSCLC	(145)
Clinical trial: NCT03835949	Atezolizumab	TJ004309 (anti-CD73 antibody)	- Ongoing Phase I trial investigating the combination in patients with advanced or metastatic cancer	
Animal study	Anti-PD-1 (RMP1-14) and anti-PD-L1 (10F.9G2) monoclonal antibodies	Trametinib (MEK inhibitor)	- Inhibition of MEK1/2 promoted YAP degradation in NSCLC - The drug combination was shown to produce synergistic anticancer effect and prolong survival of NSCLC tumor-bearing mice	(146, 147)
Animal study	Anti-PD-1 monoclonal antibodies	CDK9 inhibitor (MC180295)	- CDK9 promotes YAP-driven transcription of its downstream oncogenic effectors - MC180295 sensitizes NSCLC to anti-PD-1 antibody in C57Bl/6 mouse model	(148, 149)

CR, complete response; ORR, objective response rate; OS, overall survival; PFS, progression free survival.

As discussed above, the major Hippo regulator YAP plays critical role in regulating tumor immunity and PD-L1 expression (83–88). It follows that therapies targeting YAP may potentially enhance the efficacy of anti-PD-1/PD-L1 ICIs in EGFR-mutant NSCLC. A few small molecule compounds or drugs, including dasatinib, JQ1, norcantharidin, MLN8237 and dobutamine, have been shown to inhibit YAP (150). Further investigation is needed to verify the beneficial effect of combining YAP inhibitors with anti-PD-1/PD-L1 ICIs for treating EGFR TKI resistant NSCLC. Apart from inhibiting YAP, the modulation of YAP-related oncogenic pathways may also be evaluated. Inhibition of MEK1/2 is known to promote YAP degradation in NSCLC (146) (**Table 5**). Recently, the combination of MEK inhibitor and anti-PD-1/PD-L1 antibodies have been shown to produce synergistic anticancer effect and prolong survival of NSCLC tumor-bearing mice (147). On the other hand, cyclin-dependent kinase 9 (CDK9) is a key mediator promoting YAP-driven transcription of its downstream oncogenic effectors (148). Therefore, CDK9 inhibitors, such as dinaciclib and seliciclib, may be evaluated for potentiation of PD-1/PD-L1 blockade therapy. In fact, a recent animal study has shown that a highly selective CDK9 inhibitor (MC180295) sensitizes cancer cells to anti-PD-1 antibodies (149).

The combination of a few novel immune modulating agents and PD-1/PD-L1 ICIs have also been investigated. Eftilagimod alpha (IMP321) is a recombinant LAG-3Ig fusion protein that binds to MHC class II to activate antigen presenting cell and CD8 T-cell. The increase in activated T cells by IMP321 could potentially reduce the number of non-responders to pembrolizumab. Pilot results from the TACTI-002 trial showed that the combination of IMP321 and pembrolizumab achieved an ORR of 47% in advanced NSCLC (145) (**Table 5**). Activation of EGFR is associated with overactivation of Tregs. To this end, CD36 is known to make Tregs more adaptable to TME by serving as a metabolic modulator (151). Forced expression of CD63 by genetic approach in Tregs was shown to suppress tumor growth and enhance the antitumor efficacy of PD-1 therapy (151). CD73 is a cell surface nucleotidase, which catalyzes the hydrolysis of AMP into adenosine and phosphate. CD73-generated adenosine plays a critical role in tumor immunoescape (152). In an ongoing clinical trial (NCT03835949), the combination of an anti-CD73 drug (TJ004309) and atezolizumab is investigated in patients with advanced or metastatic cancer (153).

### Local Co-Treatments

Besides the aforementioned systemic combination treatment for potentiating immunotherapy, a few local treatment options have also been investigated. These include thermal therapies, radiotherapy and minimal invasive intratumoral therapy.

#### Immunomodulation by Local Thermal Ablation of Cancer

Thermal ablation has been used for the management of localized tumors for patients not eligible for surgical resection. A growing body of evidence suggests that thermoablation could modulate both adaptive and innate immunity (154). However, the induced immune responses are mostly weak and not sufficient for the eradication of tumors or durable prevention of disease progression. In recent years, the combination of thermal ablation and ICIs therapy have been evaluated with promising results. Shi et al. reported that radiofrequency ablation (RFA) treatment of liver metastases increased not only T cell infiltration but also PD-L1 expression in primary human colorectal tumors (155). Using mouse tumor models, RFA treatment of one tumor was found to initially enhance a strong T cells mediated immune response in tumor. However, the tumor quickly overcame the immune responses by inhibiting CD8 and CD4 T cell function, subsequently driving a shift to higher regulatory T cell to Teff ratio (155). Importantly, the combination of RFA and anti-PD-1 antibodies was found to significantly enhance T cell immune responses and lead to prolonged animal survival (155).

#### Radiotherapy to Induce ICD

Radiotherapy is commonly used in cancer therapy to achieve a local control of the irradiated target tumor lesions regardless of clinical stage. Numerous preclinical studies have demonstrated that radiotherapy could activate anti-tumor immune response. Irradiation is known to activate host immunity by triggering ICD, which is characterized by the release of DAMPs to activate dendritic cells and to prime antigen-specific T cells in a dose-dependent manner (156). Procureur et al. has recently published an excellent review about the enhancement of ICIs by radiotherapy-induced immunogenic cell death (157). Radiotherapy-induced systemic immune activation could cause shrinkage of distant tumor lesions outside the irradiated field, a phenomenon known as abscopal effect (158). In the past, abscopal effect was believed to be a very rare phenomenon. However, recent clinical data revealed that the combination of radiotherapy and anti-PD-1/PD-L1 ICIs could induce the abscopal effect (159).

On the other hand, the induction of immunosuppressive cytokines and chemokines by radiotherapy contribute to immunosuppressive reactions (160). The PD-1/PD-L1 axis is one of the key factors in cancer immune escape induced by radiotherapy. Moreover, upregulation of PD-L1 expression has been reported in NSCLC patients who have undergone radiotherapy with or without chemotherapy as preoperative treatment (161). In addition, EGFR signaling after irradiation leads to PD-L1 upregulation *via* the IL-6/JAK/STAT3 pathway (162). As anti-PD-1/PD-L1 antibodies could relieve this immunosuppression, it makes sense to combine PD-1/PD-L1 ICIs and radiotherapy (161).

The phase III PACIFIC trial (NCT02125461) provided the most remarkable clinical data to support the combination of radiotherapy and anti-PD-1/PD-L1 ICIs. In this study, progression-free survival was significantly prolonged by prescribing durvalumab as a consolidation therapy after concurrent chemoradiotherapy as compared with placebo (51). Based on this trial, durvalumab following chemoradiotherapy has been approved for the treatment of NSCLC by the US Food and Drug Administration in 2018.

#### Minimal Invasive Intratumoral Injection of ICD Inducer

Local immunotherapies such as the intratumoral injection of oncolytic compounds have been used to reinstate and enhance systemic anticancer immune responses. A recent animal study reported the use of local immunotherapy to sensitize the tumor to subsequent immune checkpoint blockade (163). LTX-401 is an oncolytic peptide designed for local immunotherapy. The sequential LTX-401 treatment combined with double checkpoint inhibition of PD-1 and CTLA-4 exhibited strong antineoplastic effects on both the primary lesions and distant tumors (163).

## Conclusion

In this review, we summarize the recent investigations about the use of PD-1/PD-L1 blockade therapy in EGFR-mutated NSCLC. The underlying mechanisms leading to the inferior clinical efficacy of PD-1/PD-L1 inhibitors in EGFR-mutated NSCLC and new strategies for its potentiation are discussed. Currently, the NCCN guidelines do not recommend immunotherapy for treating NSCLC patients carrying EGFR mutations. The combinations of PD-1/PD-L1-based immunotherapy and several other treatment modalities are under active investigation in clinical trials. While outcomes of these trials are immature, the optimal sequence, schedule and dosing remain to be determined. Moreover, the possible risk of combined toxicity pose a major challenge for the drug combinations. Therefore, a thorough investigation about the mechanism of action and risks associated with drug combinations is needed. It will help identify specific patient population that can benefit from the drug combination, predict the likelihood of toxicities, and guide dosing/administration sequencing and clinical monitoring consideration.

## Author Contributions

All authors contributed to the article and approved the submitted version.

## Conflict of Interest

The authors declare that the research was conducted in the absence of any commercial or financial relationships that could be construed as a potential conflict of interest.
